# A validated SSAM-FEA framework for the rat knee reproduces varus-induced contact redistribution in a meniscus-deficient setting

**DOI:** 10.3389/fbioe.2026.1741621

**Published:** 2026-05-22

**Authors:** Ke Lu, Yu-Rong Tao, Shao-Han Guo, Chong Li, Xue-Lian Gu, Bo Chen

**Affiliations:** 1 Department of Orthopedics, Affiliated Kunshan Hospital of Jiangsu University, Suzhou, Jiangsu, China; 2 School of Health Science and Engineering, University of Shanghai for Science and Technology, Shanghai, China; 3 Department of Orthopedics, Kunshan Hospital Affiliated to Nanjing University of Chinese Medicine, Suzhou, Jiangsu, China; 4 Department of Orthopaedics, Shanghai Key Laboratory for Prevention and Treatment of Bone and Joint Diseases, Shanghai Institute of Traumatology and Orthopaedics, Ruijin Hospital, Shanghai Jiao Tong University School of Medicine, Shanghai, China

**Keywords:** biomechanics, finite element analysis, rat knee joint, statistical shape and appearance model, tibiofemoral joint

## Abstract

**Purpose:**

To characterize tibiofemoral contact mechanics in a rat knee under progressive varus loading, evaluate whether a statistical shape and appearance model (SSAM) can reproduce specimen-specific biomechanical behavior, and assess whether this meniscus-deficient computational-experimental framework can serve as a controlled preclinical platform for studying load redistribution.

**Methods:**

Three-dimensional rat knee models were reconstructed from micro-CT images of 10 hind limbs from 5 male Sprague Dawley rats. Each limb model was alternately designated as the target, while the remaining limb models served as the training dataset for principal component analysis (PCA) to develop the SSAM. Finite element analysis (FEA) was used to assess maximum contact pressure and contact area in the medial and lateral compartments under simulated standing posture at 0%, 50%, and 100% varus loading. Because the finite element model did not include the menisci, experimental validation was performed in a matched meniscus-deficient setting using a custom biomechanical testing apparatus. Analysis of variance (ANOVA) was used to compare biomechanical outcomes among loading conditions and model types.

**Results:**

Both FEA and experimental results demonstrated consistent patterns in maximum contact pressure and contact area across varus loading levels. Compared with 0% varus, 50% and 100% varus loading increased medial maximum contact pressure and contact area by up to 0.55 MPa and 1.28 mm^2^, respectively. The lateral compartment showed reductions, with maximum decreases of 0.43 MPa and 1.76 mm^2^. No statistically significant differences were detected between the specimen-specific FEA models and the corresponding SSAM-predicted models across the tested loading conditions (P > 0.05).

**Conclusion:**

Progressive varus loading shifted contact mechanics toward the medial compartment in both FEA and experimental tests, and the SSAM reproduced these trends with good agreement to specimen-specific models. These findings support the use of the present SSAM-FEA framework as a controlled biomechanical tool for investigating varus-induced contact redistribution in a rat meniscus-deficient knee. However, because the model excludes the menisci, uses simplified material assumptions, and applies static loading, the results should be interpreted primarily within a post-meniscectomy biomechanical context rather than as a direct surrogate for naturally occurring human knee osteoarthritis.

## 1 Introduction

Knee osteoarthritis (KOA) affects over 300 million individuals worldwide, representing the most common cause of disability among adults and imposing an annual economic burden exceeding $460 billion in all-cause medical costs in the United States alone (Long et al., 2022; Lo et al., 2021). This degenerative joint disease fundamentally alters the biomechanical environment of the knee, with varus malalignment serving as both a risk factor and a consequence of disease progression. The mechanical basis of KOA pathogenesis centers on aberrant load distribution: varus deformity shifts the knee’s load-bearing axis medially, concentrating excessive stress on the medial tibiofemoral compartment—which becomes affected up to ten times more frequently than the lateral compartment (Jones et al., 2013). This sustained mechanical overload initiates a degenerative cascade that exceeds chondrocytes’ homeostatic capacity, perpetuating cartilage breakdown through mechanobiological pathways involving inflammatory mediators, matrix metalloproteinases, and altered cellular metabolism (Bader et al., 2011; Goldring and Goldring, 2010). Understanding these biomechanical drivers has become paramount for developing mechanistically-informed therapeutic strategies, as traditional pharmacological approaches have shown limited success in modifying disease progression.

Animal models, particularly rodent models such as Sprague Dawley rats, are widely used to investigate KOA-related biomechanical changes because they are practical, experimentally tractable, and compatible with controlled induction protocols (Rajalekshmi and Agrawal, 2025). However, translation from preclinical models to human disease remains challenging, in part because few quantitative frameworks link specimen-level anatomy to experimentally validated joint mechanics (Mann et al., 2020). Finite element analysis (FEA) combined with statistical shape and appearance models (SSAMs) offers a potentially useful approach for specimen-informed biomechanical analysis, reducing the need for repeated destructive testing while enabling controlled parametric evaluation (Benos et al., 2020). Yet current preclinical computational knee models often lack rigorous experimental validation and may not report anatomy, material properties, and boundary conditions with sufficient clarity to support reproducibility or careful interpretation (Halloran et al., 2023). Accordingly, there remains a need for a validated framework that can reproduce contact-mechanics redistribution under controlled loading conditions in the rat knee.

**FIGURE 1:**
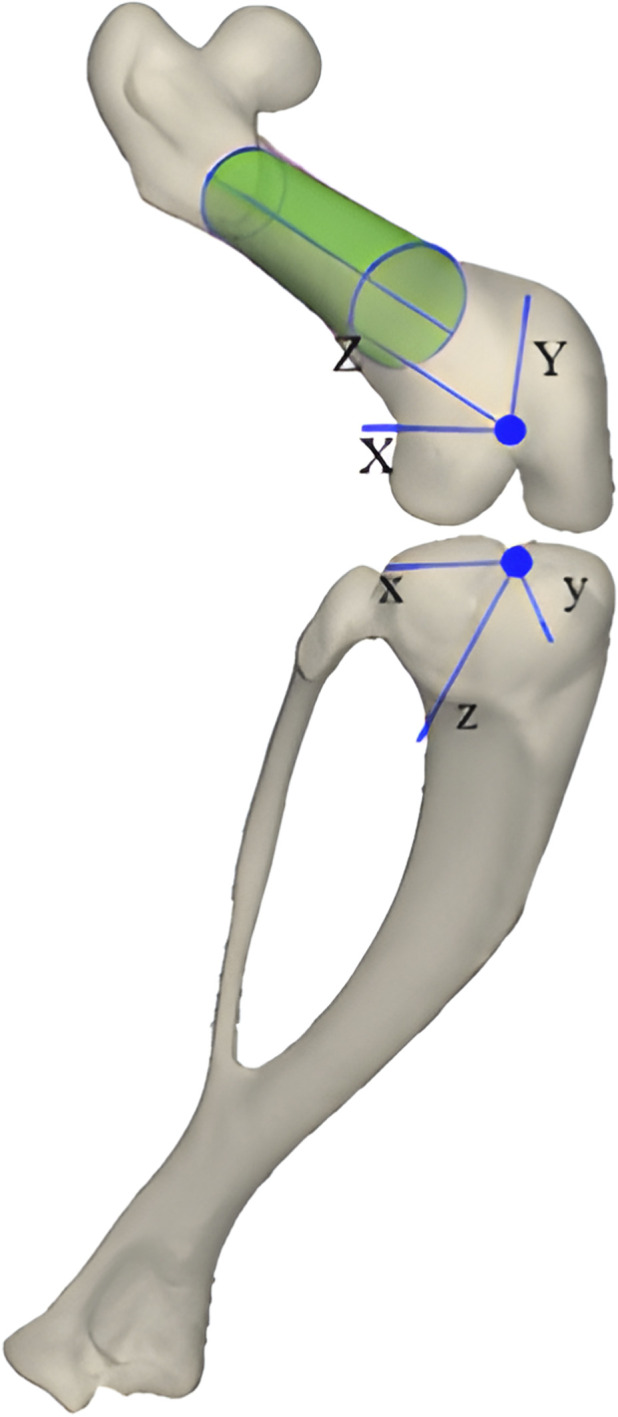
Definition of the tibiofemoral joint coordinate system.

To address this gap, we developed and experimentally validated an SSAM-FEA platform for the rat knee joint to predict tibiofemoral contact mechanics under progressive varus loading. The tibiofemoral joint coordinate system used in this study is shown in Figure 1. The primary objective of this study was to establish a computational-experimental framework that could reproduce specimen-specific contact pressure and contact area with reasonable accuracy in a controlled preclinical setting. Because the finite element model did not include the menisci and the experimental validation was performed after meniscal removal, the present work was designed to represent a meniscus-deficient, post-meniscectomy-like biomechanical scenario rather than the full physiological condition of the intact knee. Within this scope, we hypothesized that the SSAM-FEA platform would reproduce the direction and magnitude of contact-mechanics redistribution under increasing varus loading and thus provide a useful basis for future biomechanical investigations of load-induced joint degeneration in the rat knee.

## 2 Materials and methods

### 2.1 Image data acquisition

All animal procedures were approved by the Experimental Animal Ethics Committee of Kunshan First People’s Hospital (approval No. KSPH-IACUC-2025004) before study initiation. Five healthy male Sprague Dawley (SD) rats (2 months old, approximately 300 g) were euthanized via cervical dislocation. All rats were anesthetized using isoflurane (2%–3% concentration via inhalation) through a face mask until they lost voluntary movement and showed no response to external stimuli. The depth of anesthesia was closely monitored to ensure they were fully anesthetized before proceeding. Once fully anesthetized, euthanasia was performed by cervical dislocation, where the neck was quickly and firmly displaced to ensure immediate and painless death. This procedure adhered to ethical guidelines and minimized animal suffering. The hind limbs were disarticulated at the hip joint, and surrounding soft tissues, including skin and muscle, were carefully removed while preserving the femur, tibia-fibula, patella, and joint capsule. Each hind limb was imaged using a high-resolution micro-computed tomography (micro-CT) scanner (Skyscan 1,172, Bruker, Belgium) with a voxel resolution of 18 μm, operating at 49 kV and 176 μA. Scanning time was approximately 50 min per sample, and the resulting images were stored in DICOM format. All available hind limbs included in the final study cohort were analyzed, and no additional exclusions were applied after imaging and allocation of the final study specimens. Because the present study used the complete set of available hind-limb models in an exploratory limb-level design, no *a priori* sample-size calculation or formal power analysis was performed. Randomization was not applied to specimen allocation, as leave-one-out validation was performed on the full dataset. Blinding was not performed during model reconstruction, finite element analysis, experimental testing, or outcome assessment.

### 2.2 3D model reconstruction and SSAM establishment

**FIGURE 2:**
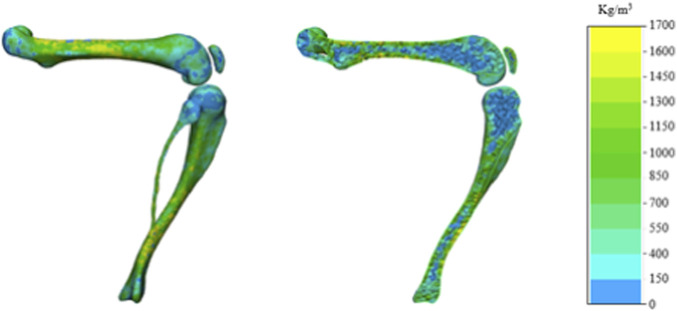
Mesh modeling of the knee joint with assigned bone density material.

Micro-CT DICOM images were imported into Mimics 21.0 (Materialise, Belgium) for segmentation. The bone regions of the distal femur and proximal tibia-fibula were identified using thresholding and masking techniques. Following manual refinement of the segmented regions, three-dimensional (3D) models of the knee joint were reconstructed. To enhance surface continuity, smoothing and wrapping operations were applied. For uniformity, each left knee model was mirrored to generate a corresponding right knee model, ensuring that all final models represented right-side knees. The reconstructed 3D models were then imported into 3-matic 15.0 (Materialise, Belgium) for meshing. Representative mesh models with assigned bone-density material properties are shown in Figure 2. An average element size of 0.3 mm was used for the femur and tibia-fibula, and 0.2 mm for the patella.

The rotational center of the rat knee, defined as the midpoint between the medial and lateral femoral epicondyles, was established as the origin of a joint coordinate system. The knee was modeled as a hinge joint with a single degree of freedom, permitting flexion and extension (Orozco et al., 2022). In an upright posture with 80° knee flexion (typical standing configuration for rats), this tibiofemoral alignment corresponds to the maximum weight-bearing contact (Das Neves Borges et al., 2014). A local orthogonal coordinate system was constructed as follows: the X-axis was aligned with the line connecting the femoral epicondyles (oriented laterally), the Z-axis was set perpendicular to the tibial plateau and directed from the femur to the tibia, and the Y-axis was defined as the cross-product of the X- and Z-axes (Grood and Suntay, 1983; Wu et al., 2002). This joint coordinate system was subsequently employed to define boundary conditions and apply mechanical loads in the finite element analyses. Using the reconstructed models, a statistical shape and appearance model (SSAM) of the rat knee was constructed. All 3D models (femur, tibia-fibula, patella) were co-registered via deformable mesh registration to establish nodal correspondence across the dataset.

Principal component analysis (PCA) was applied to the aligned knee joint models to identify the principal modes of anatomical variation. The resulting SSAM generated new knee joint geometries by varying PCA coefficients within a defined range (Clouthier et al., 2019). In this study, shape variations were constrained within ±3 standard deviations (SD) from the mean along each principal component axis, and random resampling was performed 20 times within this range for each principal component. For validation, a leave-one-out procedure was performed at the limb level: each hind limb model was sequentially designated as the target, while the remaining nine limb models served as the training set. Because the dataset comprised bilateral hind limbs from five rats, the contralateral limb of the target animal was retained in the training set during each validation iteration. In preprocessing, left knees were mirrored to a right-sided representation to standardize model orientation across specimens. Accordingly, the present validation design should be interpreted as a limb-level framework with normalized laterality rather than as a laterality-stratified analysis. The SSAM was then fitted to predict the geometry of the target model, and the predicted model was compared with the corresponding original model to evaluate geometric accuracy. Because the modeling cohort comprised 10 hind limbs from only 5 male rats of a single age group, the present dataset captures only limited biological diversity and should be interpreted as a limb-level cohort rather than a broadly representative population sample.

### 2.3 Finite element analysis of varus loading

**FIGURE 3:**
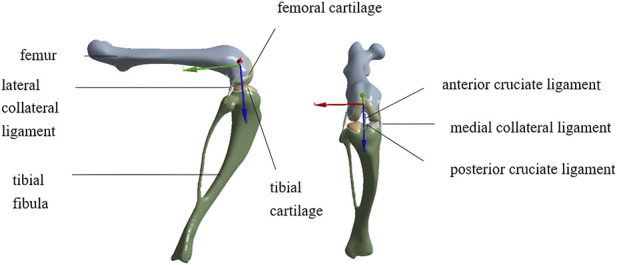
Finite element model of the rat hindlimb in the meniscus-deficient loading configuration.

The volumetric meshes of the rat knee models were imported into ANSYS Workbench 2020 (ANSYS Inc., United States) for finite element analysis. The finite element model of the rat hindlimb in the meniscus-deficient loading configuration is shown in Figure 3. Articular cartilage was represented by 0.25 mm thick hexahedral layers over the distal femoral condyles and proximal tibial plateau. For computational efficiency and consistency across specimens, cartilage was modeled as a homogeneous, isotropic, linear elastic material with a Young’s modulus of 6 MPa and a Poisson’s ratio of 0.49 (Ayobami et al., 2022). We acknowledge that this representation does not capture the viscoelastic, depth-dependent, and anisotropic behavior of native cartilage tissue, but it was adopted here to provide a standardized modeling framework for comparative analysis across loading conditions.

Bone tissue was assigned heterogeneous material properties using a literature-based, rat-specific density-modulus relationship. Following Cory et al., the compressive modulus of rat bone was calculated as E = 8362.8 ${\left(\rho_{EQUIV}\right)}^{2.56}$ , where $\rho_{EQUIV}$ denotes micro-CT-based equivalent mineral density and E is expressed in MPa (Cory et al., 2010). Material assignment was implemented during finite element preprocessing in ANSYS Workbench 2020 after geometric reconstruction and meshing in Mimics 21.0 and 3-matic 15.0. This approach enabled spatially heterogeneous stiffness assignment throughout the femur and tibia while maintaining a consistent material-definition strategy across specimens. Because the present study was designed primarily as a comparative biomechanical investigation under controlled loading conditions, this literature-based material assignment was used to provide heterogeneous stiffness representation rather than specimen-specific quantitative calibration of bone tissue properties. All bone tissues were assumed to be isotropic and linearly elastic.

The knee joint model incorporated four major ligaments, including the anterior cruciate ligament (ACL), posterior cruciate ligament (PCL), medial collateral ligament (MCL), and lateral collateral ligament (LCL). Each ligament was represented as a tension-only spring element spanning its anatomical attachment points on the bones (Gantoi et al., 2013). Based on previously reported stiffness ranges (Race and Amis, 1994; Woo et al., 1991), the ACL and PCL were each assigned a stiffness of 35 N/mm with 5% pretension, whereas the MCL and LCL were assigned 20 N/mm with a 4% pretension. This simplified representation was selected to preserve the major restraining function of the ligaments in the model, although it does not capture nonlinear stiffness, anisotropy, or time-dependent behavior observed in native ligament tissue.

Contact interactions were defined to simulate joint articulation between cartilage surfaces. The femoral cartilage was rigidly bonded to the femur, and the tibial cartilage was similarly bonded to the tibia, ensuring no relative motion at the bone-cartilage interfaces. At the tibiofemoral cartilage-cartilage interface, a frictionless sliding contact was applied, using a zero friction coefficient to mimic the low-friction behavior of healthy articular cartilage (Forster and Fisher, 1996). To enhance contact detection accuracy and avoid premature separation during analysis, a small pinball contact radius of 0.1 mm was specified. This setting helps prevent numerical convergence issues that may arise from minor initial gaps between opposing cartilage surfaces. Boundary and loading conditions were designed to represent a simplified standing-like configuration of the rat hind limb. The distal end of the tibia-fibula was fully fixed, and the knee joint was positioned at 80° of flexion, which has been reported as a representative weight-bearing posture in rats. A vertical compressive load of 2.94 N was applied downward at the femoral head to represent a standardized nominal axial load approximating one body weight for a 300 g rat. A standardized nominal load was used instead of specimen-specific loading so that between-specimen comparisons would primarily reflect differences in varus-mediated load redistribution rather than small differences in body mass. Under neutral alignment (0% varus), this load was distributed between the medial and lateral tibiofemoral compartments. Varus loading conditions were introduced by progressively redistributing the load from the lateral to the medial compartment. In the 50% varus condition, the medial compartment bore 50% more load and the lateral compartment 50% less load than in the neutral condition. In the 100% varus condition, the load was concentrated on the medial compartment, while the lateral compartment was unloaded. The total applied force remained 2.94 N in all cases.

This loading strategy was selected to provide a controlled and comparable mechanical environment across specimens and to isolate the effect of varus-mediated load redistribution on tibiofemoral contact mechanics. However, it represents a static simplification and does not account for time-varying gait loads, changing flexion angles, or lubrication-related effects that occur during physiological locomotion. The femur was allowed to translate until mechanical equilibrium was reached, and the main output parameters were peak contact pressure and total contact area on the medial and lateral cartilage surfaces.

### 2.4 Experimental validation of varus loading

**FIGURE 4:**
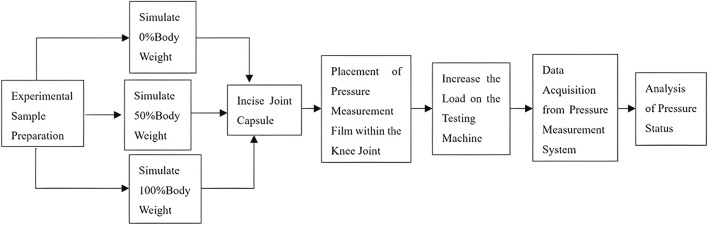
Flow chart of different varus load.

**FIGURE 5:**
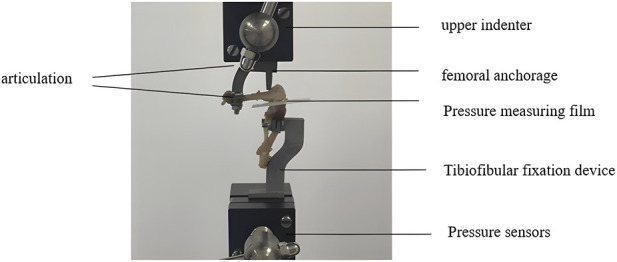
Experimental test setup for rat knee joint varus load testing.

A custom-designed biomechanical testing platform was developed to validate the FEA results. The overall workflow of the different varus loading procedures is summarized in Figure 4, and the experimental test setup for rat knee joint varus load testing is shown in Figure 5. The apparatus enabled precise mounting of rat knee specimens at 80° flexion and the application of controlled varus loads. Thawed rat knees, including the intact femur and tibia, were fixed within the testing device using clamps that maintained the prescribed alignment. The specimens used for experimental validation were obtained from the same imaged cohort used for reconstruction of the computational models. The setup allowed adjustment of femoral varus angulation to 3° and 6°, corresponding to the 50% and 100% varus loading conditions, respectively, relative to neutral alignment. A loading rod was positioned over the femoral supracondylar region to deliver compressive force. To ensure consistency between the experimental measurements and the finite element model, the joint capsule was opened, and the menisci were removed before pressure testing. Accordingly, the experimental validation represented a matched meniscus-deficient condition rather than the intact physiological knee. A thin pressure-sensitive film (Prescale®, Fujifilm, Japan) was inserted between the femoral and tibial cartilage surfaces to record the contact stress distribution.

A static axial load of 2.94 N was applied vertically using an electronic universal testing machine for 5 s under each configuration (neutral, 3° varus, and 6° varus). This standardized loading protocol was selected to mirror the simplified finite element conditions and to enable direct comparison between computational and experimental results, although it does not replicate the dynamic loading pattern of gait. Each knee underwent three repeated tests per loading condition to ensure measurement consistency. Following each test, the pressure-sensitive film was scanned and analyzed using a calibration curve to quantify both the contact area and peak pressure. For each trial, the contact areas of the medial and lateral compartments (identified as stained regions on the film) and the peak pressures (corresponding to the highest intensity regions) were recorded.

To enable direct comparison with the FEA, a femoral varus angle of 3° in the experimental fixture was designated as equivalent to the 50% varus load condition, while 6° represented the 100% varus condition. For each compartment, the experimentally measured peak pressures and contact areas were averaged across three repeated trials. Statistical comparisons among model types and loading conditions were performed as described in Section 2.5.

### 2.5 Statistical analysis

All statistical analyses were performed using IBM SPSS Statistics 27.0 (IBM Corp., Armonk, NY, United States). Data used for statistical analysis were recorded at the knee level, and tabulated results are presented as group means. The normality of the data distribution was assessed using the Shapiro–Wilk test. Comparisons of biomechanical outcomes among loading conditions and model types were performed using one-way analysis of variance (ANOVA), with analyses conducted separately for peak contact pressure and contact area in the medial and lateral compartments. When appropriate, *post hoc* pairwise comparisons were performed using Tukey’s multiple comparisons test. A P value < 0.05 was considered statistically significant.

## 3 Results

**FIGURE 6:**
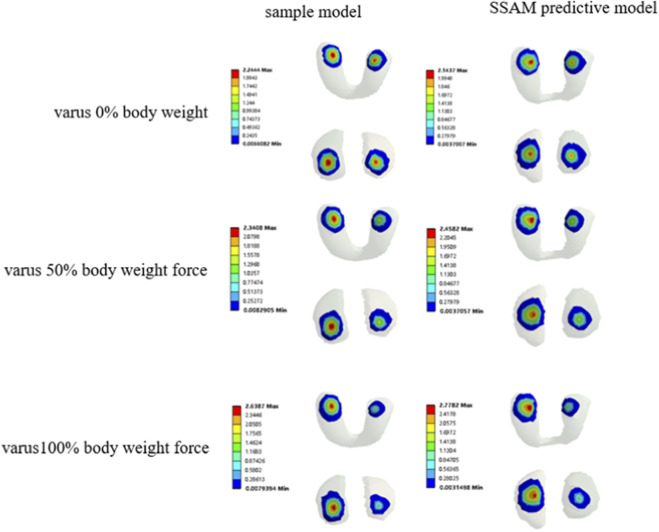
Contact pressure distribution in the tibiofemoral joint cartilage.

The FEA simulations and corresponding experimental results exhibited consistent trends in how varus loading influences tibiofemoral joint contact in the rat knee. Color maps illustrating the FEA-derived contact pressure distributions under 0%, 50%, and 100% varus conditions are presented in Figure 6. Under neutral loading (0% varus), contact pressure was evenly distributed between the medial and lateral tibial cartilage surfaces. As varus loading increased, contact stress progressively shifted toward the medial compartment. In both the original sample models and the SSAM-predicted models, higher pressure concentrations were observed on the medial tibial plateau, while pressure on the lateral side decreased. The contact pressure patterns predicted by the SSAM closely resembled those of the corresponding sample models, suggesting that the SSAM accurately captured the critical geometric features that influence joint contact mechanics.

Table 1 summarizes the peak contact pressures in the medial and lateral compartments under different varus loading conditions. In the medial compartment, mean peak pressure increased progressively with increasing varus loading in the original sample model, the SSAM-predicted model, and the experimental measurements. In contrast, lateral peak pressure decreased as load redistribution shifted toward the medial side. The numerical values obtained from the SSAM-predicted models were generally comparable to those of the specimen-specific models and the experimental measurements. One-way ANOVA indicated no statistically significant differences in peak pressure between the SSAM-predicted models and the specimen-specific models under the tested loading conditions.

**TABLE 1:** Peak contact pressure in the medial and lateral tibiofemoral compartments under different varus loading conditions.

Loading condition	Model type	Medial peak pressure (MPa, mean)	Lateral peak pressure (MPa, mean)
0% varus	Original sample FEA	1.77	1.66
​	SSAM-predicted FEA	1.80	1.64
​	Experimental	1.77	1.77
50% varus	Original sample FEA	2.06	1.45
​	SSAM-predicted FEA	2.15	1.42
​	Experimental	2.05	1.45
100% varus	Original sample FEA	2.30	1.25
​	SSAM-predicted FEA	2.35	1.21
​	Experimental	2.40	1.10

Values are presented as group means. Experimental values were obtained by averaging three repeated measurements for each knee under each loading condition before group-level statistical analysis.

**FIGURE 7:**
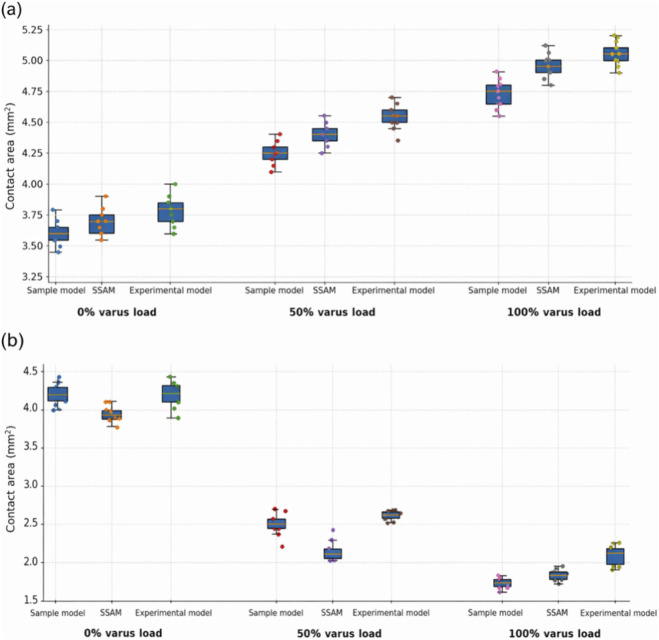
Contact area in the medial **(a)** and lateral **(b)** tibiofemoral compartments under neutral, 50% varus, and 100% varus loading. The figure compares original sample FEA, SSAM-predicted FEA, and experimental measurements. Contact area is reported in ${mm}^{2}$ .

Figure 7 depicts the contact area measurements for the medial and lateral compartments under varying varus loading conditions. In the medial compartment, increasing varus load from 0% to 50% caused the average contact area to increase by roughly 0.63 mm^2^ (sample), 0.92 mm^2^ (SSAM), and 0.66 mm^2^ (experiment). At 100% varus, medial contact area further increased by about 1.07 mm^2^ (sample), 1.28 mm^2^ (SSAM), and 1.11 mm^2^ (experiment), relative to the neutral condition. In contrast, lateral contact areas decreased under varus: at 100% varus, lateral contact area reductions were approximately 1.37 mm^2^ (sample), 1.17 mm^2^ (SSAM), and 1.42 mm^2^ (experiment) compared to neutral. As with contact pressure, the SSAM’s predictions for contact area closely matched those of the sample models. For example, at 100% varus, the medial contact area was 8.5 mm^2^ (SSAM) versus 8.3 mm^2^ (sample), and the lateral area was 5.1 mm^2^ (SSAM) versus 5.3 mm^2^ (sample). No statistically significant differences were detected between the specimen-specific FEA models and the corresponding SSAM-predicted models under the tested loading conditions (P > 0.05).

Across the tested loading conditions, the experimental measurements showed trends similar to those observed in the specimen-specific and SSAM-predicted FEA models for both peak contact pressure and contact area.

## 4 Discussion

This study developed and experimentally evaluated an SSAM-FEA framework for the rat knee under progressive varus loading. The most important finding was that increasing varus loading consistently shifted tibiofemoral contact mechanics toward the medial compartment, resulting in increased medial peak contact pressure and contact area together with reduced loading on the lateral side. The similarity between the specimen-specific FEA models, the SSAM-predicted models, and the experimental measurements indicates that the present workflow can reproduce the overall pattern of contact redistribution in the rat knee under controlled loading conditions.

These findings support the value of the current framework primarily as a computational-experimental tool for comparative biomechanical analysis. The medial shift in joint loading observed here is consistent with the established mechanical effect of varus alignment and therefore reinforces, rather than redefines, current understanding of load redistribution in the knee. In this respect, the present study extends previous work in rat knee biomechanics that has shown altered contact stress under varus loading and degenerative changes induced by chronic load imbalance (Gardner-Morse et al., 2013; Roemhildt et al., 2013). By integrating statistical shape modeling with finite element analysis and experimental validation, the current work adds a specimen-informed modeling strategy that can be used to evaluate whether predicted geometries retain biomechanical behavior comparable to that of the original knees. This combined approach provides a stronger basis for comparative analysis than reliance on simulation or experiment alone (Anderson et al., 2011).

The numerical increase in medial peak contact pressure observed under severe varus loading was close to values reported in human studies (Segal et al., 2009). However, this similarity should be interpreted with caution. In the present context, it is more appropriate to regard that comparison as a supportive reference than as evidence that the current model establishes a direct cross-species pathogenic threshold for KOA. Most preclinical studies have shown that mechanical overloading contributes to cartilage degeneration and OA-like changes (Wang et al., 2024; Zhao et al., 2023; Zhang et al., 2022), but the present data are better understood as showing that the SSAM-FEA framework can reproduce the expected direction and approximate magnitude of load redistribution in a controlled rat knee model, rather than demonstrating a definitive translational threshold linking rat and human disease.

The interpretation of these findings must also consider the specific scope of the model. Because the finite element model did not include the menisci and the experimental validation was performed after meniscal removal, the present framework is most appropriately understood as representing a meniscus-deficient or post-meniscectomy biomechanical condition. Since the menisci are major load bearing and load-distributing structures of the knee, their absence would be expected to increase the absolute contact pressure relative to the intact joint. For this reason, the absolute values reported here should not be directly extrapolated to the normal physiological state, even though the relative trends in varus-induced load shifting remain informative for this specific loading scenario.

The simplifying assumptions applied to soft tissues should likewise be considered when evaluating the results. In this model, articular cartilage was represented as a homogeneous, isotropic, linear elastic material, and the ligaments were simplified as spring elements. These assumptions improved computational tractability and enabled standardized comparisons across specimens, but they do not capture the full viscoelastic, anisotropic, and nonlinear behavior of native joint tissues. The present framework is therefore more suitable for comparative analysis of relative contact-mechanics changes than for detailed reconstruction of tissue-level physiological behavior. The loading configuration was also intentionally simplified. A standardized static axial load applied at a fixed flexion angle provided a practical basis for direct comparison between simulation and experiment, but it does not reflect the dynamic alternating loads, changing joint kinematics, or lubrication effects that occur during gait (Kumar et al., 2018).

The anatomical dataset used to construct the SSAM was also limited, consisting of 10 hind limbs from 5 young male rats. This restricts the biological diversity represented in the model, and similarities between bilateral limbs from the same animal, together with the retention of the contralateral limb in the training set during leave-one-out validation, may reduce the effective morphological variation captured by the PCA-based framework and may also increase geometric similarity between the target and training sets. The present study should therefore be viewed as a preliminary limb-level biomechanical investigation rather than a comprehensive population-level characterization of rat knee variability. Future work should expand the sample size, include animals with broader age and sex distributions, incorporate meniscal geometry, and adopt more physiologically realistic constitutive models for cartilage and ligaments. These improvements would strengthen the physiological relevance of the model and clarify the extent to which the framework may be applied to broader questions of joint degeneration. In addition, extension of the present platform to controlled studies of alignment correction and load redistribution may provide a useful basis for future biomechanical simulation of procedures such as high tibial osteotomy (Fucentese et al., 2020) as well as for investigating how anatomy and alignment jointly influence compartmental load transfer (Van Rossom et al., 2019; Kang et al., 2018).

## 5 Conclusion

We developed and experimentally evaluated an SSAM-FEA framework for the rat knee under progressive varus loading. The results showed that increasing varus alignment consistently shifted contact mechanics toward the medial compartment, leading to higher medial peak contact pressure and contact area together with reduced loading on the lateral side. The SSAM-predicted models reproduced these trends with reasonable agreement to the specimen-specific finite element models and the experimental measurements, indicating that the proposed workflow can capture the overall pattern of varus-induced contact redistribution in the rat knee.

Within the scope of the present study, this framework provides a useful computational-experimental tool for comparative biomechanical investigation. At the same time, the current model should be interpreted within the specific conditions under which it was established, namely a meniscus-deficient, statically loaded rat knee model with simplified soft-tissue material representations and limited sample diversity. Further refinement, including incorporation of meniscal geometry, more physiologically realistic constitutive behavior, broader biological sampling, and dynamic loading conditions, will be needed before broader physiological or translational conclusions can be drawn.

## Data Availability

The raw data supporting the conclusions of this article will be made available by the authors, without undue reservation.
